# Nordalbergin Exerts Anti-Neuroinflammatory Effects by Attenuating MAPK Signaling Pathway, NLRP3 Inflammasome Activation and ROS Production in LPS-Stimulated BV2 Microglia

**DOI:** 10.3390/ijms24087300

**Published:** 2023-04-14

**Authors:** Jung Lo, Hsin-En Wu, Ching-Chih Liu, Kun-Che Chang, Po-Yen Lee, Po-Len Liu, Shu-Pin Huang, Pei-Chang Wu, Tzu-Chieh Lin, Yu-Hung Lai, Yo-Chen Chang, Yuan-Ru Chen, Sheng-I Lee, Yu-Kai Huang, Shu-Chi Wang, Chia-Yang Li

**Affiliations:** 1 Graduate Institute of Clinical Medicine, College of Medicine, Kaohsiung Medical University, Kaohsiung 80708, Taiwan; 2 Department of Ophthalmology, Kaohsiung Chang Gung Memorial Hospital and Chang Gung University College of Medicine, Kaohsiung 83301, Taiwan; 3 Graduate Institute of Medicine, College of Medicine, Kaohsiung Medical University, Kaohsiung 80708, Taiwan; 4 Department of Ophthalmology, Chi Mei Medical Center, Tainan 71004, Taiwan; 5 Department of Ophthalmology, Louis J. Fox Center for Vision Restoration, University of Pittsburgh School of Medicine, Pittsburgh, PA 15213, USA; 6 Department of Neurobiology, Center of Neuroscience, University of Pittsburgh School of Medicine, Pittsburgh, PA 15213, USA; 7 Department of Ophthalmology, Kaohsiung Medical University Hospital, Kaohsiung Medical University, Kaohsiung 80708, Taiwan; 8 Department of Respiratory Therapy, College of Medicine, Kaohsiung Medical University, Kaohsiung 80708, Taiwan; 9 Department of Urology, Kaohsiung Medical University Hospital, Kaohsiung Medical University, Kaohsiung 80708, Taiwan; 10 Department of Urology, School of Medicine, College of Medicine, Kaohsiung Medical University, Kaohsiung 80708, Taiwan; 11 Ph.D. Program in Environmental and Occupational Medicine, College of Medicine, Kaohsiung Medical University, Kaohsiung 80708, Taiwan; 12 Division of Cardiology, Department of Internal Medicine, Kaohsiung Medical University, Kaohsiung 80708, Taiwan; 13 Department of Ophthalmology, School of Medicine, College of Medicine, Kaohsiung Medical University, Kaohsiung 80708, Taiwan; 14 Department of Neurosurgery, Kaohsiung Medical University Hospital, Kaohsiung 80708, Taiwan; 15 Department of Medical Laboratory Science and Biotechnology, Kaohsiung Medical University, Kaohsiung 80708, Taiwan; 16 Department of Medical Research, Kaohsiung Medical University Hospital, Kaohsiung 80756, Taiwan; 17 Department of Biological Science and Technology, National Pingtung University of Science and Technology, Pingtung 91201, Taiwan

**Keywords:** neurodegenerative disease, neuroinflammation, nordalbergin, microglia, MAPK signaling pathways, NLRP3 inflammasome

## Abstract

Microglia-associated neuroinflammation is recognized as a critical factor in the pathogenesis of neurodegenerative diseases; however, there is no effective treatment for the blockage of neurodegenerative disease progression. In this study, the effect of nordalbergin, a coumarin isolated from the wood bark of *Dalbergia sissoo*, on lipopolysaccharide (LPS)-induced inflammatory responses was investigated using murine microglial BV2 cells. Cell viability was assessed using the MTT assay, whereas nitric oxide (NO) production was analyzed using the Griess reagent. Secretion of interleukin-6 (IL-6), tumor necrosis factor-α (TNF-α) and interleukin-1β (IL-1β) was detected by the ELISA. The expression of inducible NO synthase (iNOS), cyclooxygenase (COX)-2, mitogen-activated protein kinases (MAPKs) and NLRP3 inflammasome-related proteins was assessed by Western blot. The production of mitochondrial reactive oxygen species (ROS) and intracellular ROS was detected using flow cytometry. Our experimental results indicated that nordalbergin ≤20 µM suppressed NO, IL-6, TNF-α and IL-1β production; decreased iNOS and COX-2 expression; inhibited MAPKs activation; attenuated NLRP3 inflammasome activation; and reduced both intracellular and mitochondrial ROS production by LPS-stimulated BV2 cells in a dose-dependent manner. These results demonstrate that nordalbergin exhibits anti-inflammatory and anti-oxidative activities through inhibiting MAPK signaling pathway, NLRP3 inflammasome activation and ROS production, suggesting that nordalbergin might have the potential to inhibit neurodegenerative disease progression.

## 1. Introduction

Neurodegenerative diseases, a range of diseases affecting the central nervous system (CNS) leading to the progressive loss of neural structures and neurological functions, are among the most common causes of disability and morbidity globally [1,2]. As one of the major threats to human health, neurodegenerative diseases, including Alzheimer’s disease (AD), Parkinson’s disease and amyotrophic lateral sclerosis result in a wide range of incurable symptoms, such as motor dysfunction, memory impairment, cognitive defects and psycho-behavioral changes while causing a large unmet medical need [1,3]. AD dementia is the most common form of neurodegenerative disease and is estimated to account for 60–70% of the approximately 50 million dementia patients worldwide [3]. However, only two classes of drugs, including inhibitors of the enzyme cholinesterase and antagonists of N-methyl d-aspartate, are currently approved to treat symptoms but not to cure or prevent AD [4].

Neurodegenerative diseases are characterized by a dysregulated neuroglial microenvironment. Several cellular and molecular mechanisms are thought to be involved, including inflammatory responses, mitochondrial dysfunctions, increases in oxidative stress, altered cell signaling, neuronal apoptosis and abnormal deposition of proteinaceous materials [2,5]. Neuroinflammation refers to an inflammatory response in the CNS and is considered a hallmark of neurodegenerative diseases, with the activation of microglia being the key element of neuroinflammation [6]. Microglia are intrinsic immune cells located in the CNS, and the activation of microglia functions as an innate immune defense that is essential for protecting the brain from various pathological insults [7]. During neuroinflammation, microglia recognize injurious stimuli (e.g., lipopolysaccharide, LPS) via Toll-like receptor-4 and consequently trigger the activation of mitogen-activated protein kinases (MAPKs), including C-Jun N-terminal kinase (JNK), extracellular signal-regulated kinase (ERK) and p38 MAPK [6]. However, excessive, uncontrolled and sustained microglial activation—with losing homeostatic functions and producing increased pro-inflammatory cytokines, including tumor necrosis factor-α (TNF-α) and interleukin (IL)-6—is detrimental, and can eliminate synaptic structure of neurons, worsen neurodegenerative processes and lead to neurological dysfunction [1,2,6,7]. In addition, inflammasomes are multimeric protein complexes typically consisting of a sensor Nod-like receptor (NLR) molecule, the adaptor protein apoptosis-associated speck-like protein containing a CARD (ASC) and caspase-1 [8]. Among several inflammasome types, the NLRP3 inflammasome is a well-characterized protein complex in neurodegenerative diseases, especially in AD [9]. The NLRP3 inflammasome activation results in the secretion of caspase-1-mediated IL-1β and IL-18 by microglial cells, and is associated with the development and exacerbation of AD [10]; accordingly, the modulation of this microglia activation is considered a critical therapeutic strategy to attenuate neuroinflammation [6,7].

Recent studies focusing on the development of novel therapies for neurodegenerative diseases such as AD have proposed several therapeutic strategies, including chaperones, disease-modifying therapeutics and natural compounds [4]. Coumarins are abundantly found in many plants and are classified into several categories based on the chemical structural diversity in this family of compounds [11,12]. Coumarin derivatives possess a wide variety of biological properties, including anti-oxidant, anti-angiogenic and anti-bacterial activities, and some have been used clinically as anticoagulants (warfarin, anti-vitamin K compounds) and as a photosensitizer (methoxsalen) along with ultraviolet light in the treatment of psoriasis [11,13]. Nordalbergin, a bioactive coumarin compound isolated from the wood bark of *Dalbergia sissoo*, has been reported to exhibit potent activity in inducing the terminal differentiation of human promyelocytic leukemia HL-60 cells toward mature monocytes/macrophages [14]. Since MAPKs cascades are one of the major signaling pathways involved in numerous cellular processes, such as cell survival, cell proliferation, inflammation and cell differentiation, the inhibitory effects of some coumarins on MAPKs signaling pathways have been shown in previous studies [15,16], although the biological activities of nordalbergin remain unclear.

In this study, we investigated the anti-inflammatory effects and underlying molecular mechanisms of nordalbergin in LPS-stimulated BV2 cells. Firstly, we examined the effects of nordalbergin on the production of pro-inflammatory cytokines (TNF-α and IL-6) and a neurotoxic mediator, nitric oxide (NO), by LPS-stimulated BV2 cells. Secondly, the effects of nordalbergin on the activation of the MAPKs signaling pathway were determined. Thirdly, the effects of nordalbergin on the secretion of IL-1β and NLRP3 inflammasome activation were examined via LPS/adenosine triphosphate (ATP)-stimulated BV2 cells. Finally, the effects of nordalbergin on the production of intracellular reactive oxygen species (ROS) and mitochondrial ROS by LPS-stimulated BV2 cells were examined. Experimental results showed that nordalbergin repressed the secretion of NO, IL-6, TNF-α and IL-1β; suppressed the expression of inducible NO synthase (iNOS) and cyclooxygenase (COX-2); attenuated the activation of the MAPKs signaling pathway and the NLRP3 inflammasome; and reduced both intracellular and mitochondrial ROS production by LPS-stimulated BV2 cells.

## 2. Results

### 2.1. Nordalbergin Decreases the Secretion of NO and the Expression of iNOS and COX-2 by LPS-Stimulated BV2 Cells

**Figure 1:**
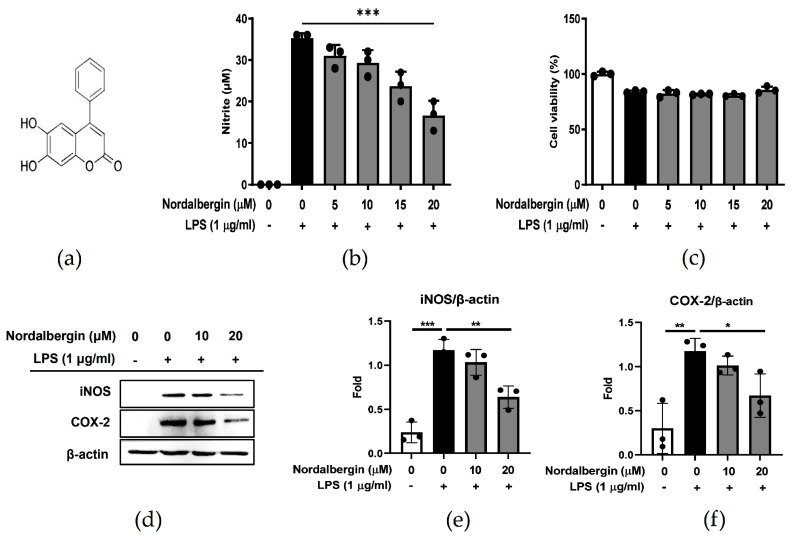
Effect of nordalbergin on the NO production, iNOS and COX-2 expression, and cell viability by LPS-stimulated BV2 cells. (**a**) The chemical structure of nordalbergin (M.W. 254.24). BV2 cells were pretreated with nordalbergin (0–20 μM) for 30 min and then primed with LPS for 24 h. (**b**) Production of NO was analyzed using Griess reagent assay. (**c**) Cell viability was detected using MTT assay. (**d**) iNOS and COX-2 expressions were measured by Western blot, while β-actin expression was used as a loading control. Quantitative results are shown in (**e**,**f**). Data are shown as mean ± standard deviation (SD) of three independent replicates (* *p* < 0.05, ** *p* < 0.01, *** *p* < 0.01).

NO is a primary mediator in modulating inflammatory responses, and is produced with the involvement of inflammation-related enzymes such as iNOS [17]. To examine whether nordalbergin affects the production of NO by LPS-stimulated microglia, BV2 cells were pretreated with nordalbergin for 30 min and then primed with LPS for 24 h. The production of NO was assessed using the Griess reagent assay. The experimental results demonstrated that nordalbergin inhibited NO production by LPS-stimulated BV2 cells, and a high concentration of nordalbergin (20 µM) significantly suppressed the production of NO (*p* < 0.001) (Figure 1a). To avoid the toxic effect of nordalbergin, the 3-(4,5-dimethylthiazol-2-yl)-2,5-diphenyltetrazolium bromide (MTT) assay was performed to examine the viability of cells after treatments with nordalbergin and LPS. According to the results, the LPS group had only slightly decreased cell viability, while nordalbergin did not affect cell survival at the concentrations of nordalbergin ≤20 µM (Figure 1b); therefore, the following experiments were performed using nordalbergin ≤20 µM. To further examine whether nordalbergin affects the expression of iNOS and COX-2 by LPS-stimulated BV2 cells, cells were pretreated with nordalbergin for 30 min and then primed with LPS for 24 h. The expression of iNOS and COX-2 was measured using Western blot. As shown in Figure 1d–f, nordalbergin significantly attenuated iNOS and COX-2 expressions by LPS-stimulated BV2 cells.

### 2.2. Nordalbergin Suppresses LPS-Induced Pro-Inflammatory Cytokine Production by Murine Microglial BV2 Cells

**Figure 2:**
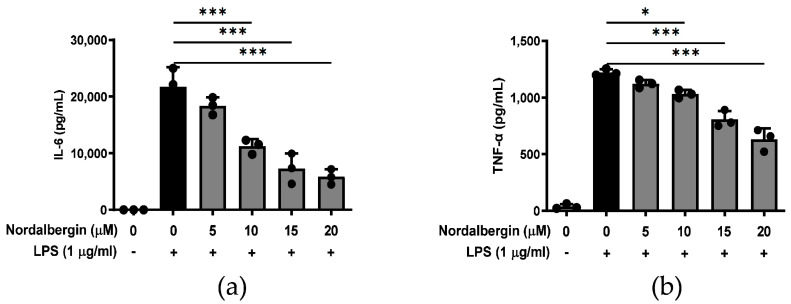
Effect of nordalbergin on the production of IL-6 and TNF-α by LPS-stimulated BV2 cells. Cells were pretreated with nordalbergin (0–20 μM) for 30 min and then primed with LPS for 24 h. Production of (**a**) IL-6 and (**b**) TNF-α was detected using ELISA. Data are shown as mean ± SD (n = 3; * *p* < 0.05, *** *p* < 0.01).

Both IL-6 and TNF-α are critical pro-inflammatory cytokines in response to LPS, and are considered to be involved in the patho-mechanisms of neurodegenerative diseases [18]. To determine the effects of nordalbergin on IL-6 and TNF-α secretion by LPS-stimulated BV2 cells, cells were pretreated with nordalbergin for 30 min and then primed with LPS for 24 h. The production of IL-6 and TNF-α was examined using the enzyme-linked immunosorbent assay (ELISA). As shown in Figure 2a,b, ≥10 μM nordalbergin significantly reduced the levels of IL-6 and TNF-α secreted by LPS-stimulated BV2 cells.

### 2.3. Nordalbergin Decreases the Phosphorylation of ERK, JNK and p38 MAPK by LPS-Stimulated Microglial BV2 Cells

**Figure 3:**
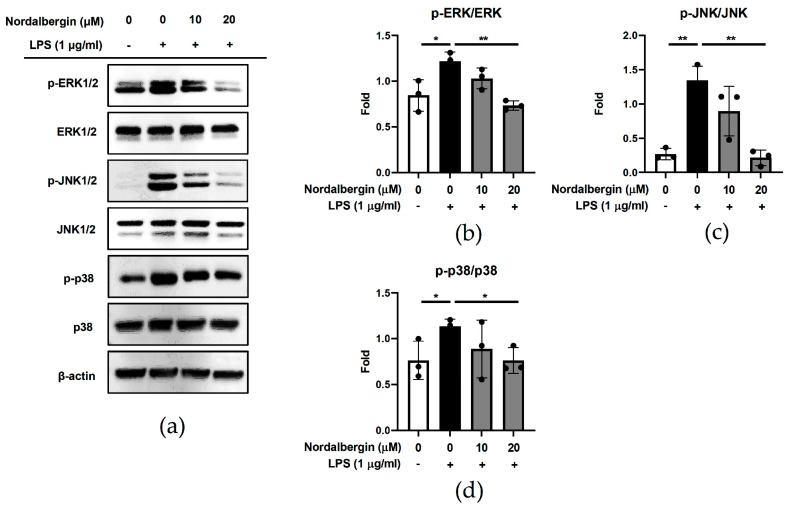
Effect of nordalbergin on the activation of MAPKs signaling pathways by LPS-stimulated BV2 cells. Cells were pretreated with nordalbergin (0–20 μM) for 30 min and then primed with LPS for 2 h. (**a**) Phospho-ERK, ERK, phospho-JNK, JNK, phospho-p38 and p38 expressions were determined using Western blot, while β-actin expression was used as a loading control. Quantitative results are shown in (**b**–**d**). Data are shown as mean ± SD (n = 3; * *p* < 0.05, ** *p* < 0.01).

MAPKs (ERK, JNK and p38 MAPK) play crucial roles in the modulation of inflammatory responses through regulating the production of pro-inflammatory mediators and cytokines by phosphorylating various proteins including transcription factors [19]. To examine the effects of nordalbergin on the MAPKs signaling pathway activation, BV2 cells were pretreated with nordalbergin for 30 min and primed with LPS for 2 h. MAPKs-related protein expressions were detected by Western blot. The experimental results showed that nordalbergin significantly reduced the phosphorylation of ERK, JNK and p38 MAPK by LPS-stimulated BV2 cells, indicating that nordalbergin significantly attenuated the activation of ERK, JNK and p38 MAPK signaling pathways under LPS stimulation (Figure 3a–d).

### 2.4. Nordalbergin Inhibits the Secretion of IL-1β and Represses the Activation of NLRP3 Inflammasome in LPS-Stimulated BV2 Cells

**Figure 4:**
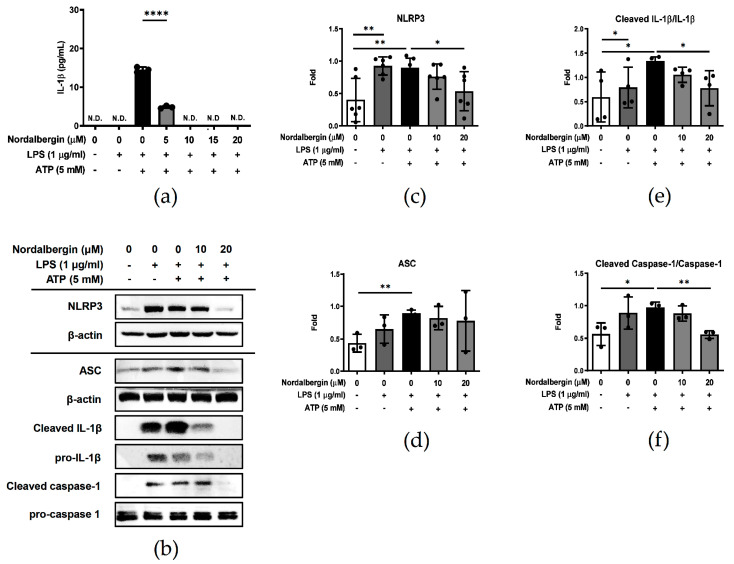
Effect of nordalbergin on the secretion of IL-1β and the activation of NLRP3 inflammasome by LPS-stimulated BV2 cells. Cells were pretreated with different concentrations of nordalbergin for 30 min and primed with LPS for 5 h following treatment of 5 mM ATP. (**a**) The secretion of IL-1β was analyzed by ELISA. (**b**) Expressions of NLRP3, ASC, cleaved-IL-1β, pro-IL-1β, cleaved-caspase-1 and pro-caspase-1 were examined by Western blot, with that of β-actin used as a loading control. The quantified results are shown in (**c**–**f**). N.D. means not detected. Data are presented as mean ± SD (n = 3–6; * *p* < 0.05, ** *p* < 0.01, **** *p* < 0.0001).

NLRP3 inflammasome plays a central role in the pathogenesis of many inflammatory disorders, including AD, by secreting high quantities of the pro-inflammatory cytokines such as IL-1β [8]. To examine the effect of nordalbergin on IL-1β secretion and NLRP3 inflammasome activation, BV2 cells were pretreated with nordalbergin for 30 min and primed with LPS for 5 h following treatment of 5 mM ATP. Cell supernatant was collected and the production of IL-1β analyzed by the ELISA, whereas the cells were lysed and the expression of NLRP3 inflammasome-associated proteins were determined using Western blot. As shown in Figure 4a, nordalbergin significantly inhibited LPS/ATP-induced IL-1β secretion by BV2 cells. Moreover, nordalbergin also suppressed the expression of NLRP3 and decreased the cleavage of both caspase-1 and IL-1β by LPS/ATP-stimulated BV2 cells (Figure 4b–f).

### 2.5. Nordalbergin Decreases Both Intracellular and Mitochondrial ROS Production by LPS-Stimulated BV2 Cells

**Figure 5:**
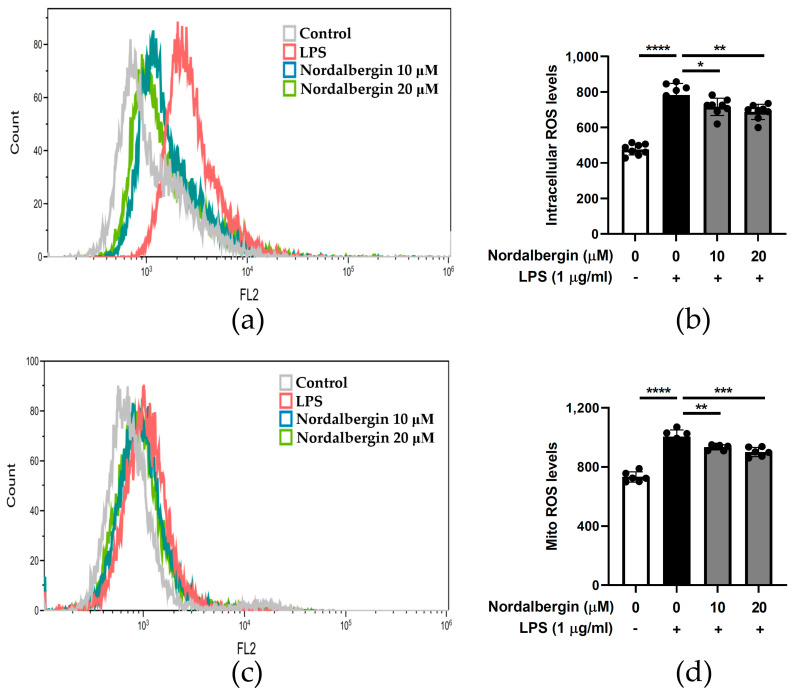
Effect of nordalbergin on the production of both intracellular and mitochondrial ROS by LPS-stimulated d BV2 cells. Cells were pretreated with various concentrations of nordalbergin for 30 min and then treated with LPS for 24 h. (**a**) Cells were stained with 1 μΜ H_2_DCFDA and the levels of intracellular ROS analyzed using flow cytometry. (**b**) The quantitative results of intracellular ROS. (**c**) Cells were stained with 5 μΜ MitoSOX red and levels of mitochondrial ROS analyzed using flow cytometry. (**d**) The quantitative results of mitochondrial ROS. Data are presented as mean ± SD (n = 6–8; * *p* < 0.05, ** *p* < 0.01, *** *p* < 0.001, **** *p* < 0.0001).

We investigated the antioxidant potential of nordalbergin by detecting the levels of intracellular and mitochondrial ROS by H_2_DCFDA and MitoSOX red staining, respectively. BV2 cells were pretreated with nordalbergin for 30 min and primed with LPS for 24 h. Then, cells were stained with 1 μΜ H_2_DCFDA or 5 μΜ MitoSOX red and measured using flow cytometry. The experimental results illustrated that both 10 and 20 µM nordalbergin significantly reduced the production of intracellular ROS by LPS-stimulated BV2 cells (Figure 5a,b). Moreover, nordalbergin also reduced the levels of mitochondrial ROS by LPS-stimulated BV2 cells (Figure 5c,d).

## 3. Discussion

Neurodegenerative diseases are prevalent in aging adults worldwide and cause serious health problems by progressive but incurable morbidity, including memory, cognitive and motor impairments [3,4,20]. Recently, alleviating excessive neuroinflammation through modulating microglial activation has become one of the new therapeutic strategies in the treatment of neurodegenerative diseases [6,7]. In the present study, we have first demonstrated that nordalbergin effectively suppressed the inflammatory responses in LPS-stimulated murine microglial BV2 cells by decreasing the secretion of inflammatory mediators and cytokines, while further suppressing the production of intracellular and mitochondrial ROS. Collectively, nordalbergin might possess the potential to exert anti-inflammatory and anti-oxidative effects and inhibit neuroinflammation through MAPK signaling pathways and NLRP3 inflammasome activation.

Natural and synthetic coumarins have been reported in a growing number of studies as therapeutic agents with a wide range of pharmacological activities including antibacterial [21], anticoagulant [12], antioxidant [22], antiangiogenic [23] and anticancer effects [24]; some coumarin derivatives are commercially available drugs [11,13]. A review article of studies on coumarins conducted in the last two decades concluded that coumarins are potent anti-inflammatory agents [11]. A previous experimental study demonstrated the effects of coumarin derivatives on various anti-inflammatory signaling pathways, such as Toll-like receptors, Janus Kinase/Signal Transducer and Activator of Transcription (JAK/STAT), inflammasomes, MAPK, NF-κB and transforming growth factor-β/small mothers against decapentaplegic (TGF-β/SMAD) pathways [11]. As a coumarin compound, nordalbergin has not been well-investigated and only one study has reported that nordalbergin regulates the differentiation of human promyelocytic leukemia cells (HL-60) toward mature monocyte/macrophage [14]. In this study, we have first demonstrated that nordalbergin has both anti-inflammatory and antioxidant activities on the inhibition of inflammatory responses and ROS production in LPS-stimulated microglial cells through attenuating MAPK signaling pathways and NLRP3 inflammasome activation, suggesting its beneficial effect in attenuating neuroinflammation.

Neurodegenerative diseases are difficult to treat because of their heterogeneous nature and complex pathological mechanisms, and neuroinflammation has been considered as a critical cellular and molecular characteristic [6,7]. Under a pathological challenge, activated microglia initiate a series of biochemical cascades as inflammatory responses, resulting in increased levels of pro-inflammatory and cytotoxic mediators and the subsequent breakdown of the extracellular matrix, cellular integrity, blood-brain barrier (BBB) and neuronal cell degeneration [6]. Among the associated inflammatory molecules, iNOS is one of the primary regulators of the inflammatory response, which expresses in glial cells, macrophages and neutrophils, and is produced after induction by inflammatory cytokines or endotoxins [25,26]. iNOS is responsible for the biosynthesis of NO and exhibits neurotoxicity on neurodegenerative diseases when generating higher concentrations of NO [25].

Moreover, the local release of pro-inflammatory cytokines, including TNF-α and IL-6, especially by myeloid cells, would cause the recruitment of leukocytes across the BBB, increasing inflammatory responses and consequently leading to neuroinflammation [27]. TNF-α and IL-6 have been found to be involved in the pathogenesis of neurodegenerative diseases, and TNF-α inhibitors are thought to have therapeutic potential in AD by improving cognitive performance [28,29]. In the present study, our results showed that nordalbergin inhibited the production of NO; decreased the expression of iNOS; and suppressed the production of pro-inflammatory cytokines (TNF-α and IL-6) in LPS-stimulated murine microglial cells, suggesting that nordalbergin might have potential in alleviating neuroinflammation by attenuating neurotoxic effects and inflammatory effects.

The MAPK family and its associated downstream transcription factor, nuclear factor-κB (NF-κB), regulate several cellular processes, such as cell proliferation, differentiation, migration and apoptosis [30,31]. Among the MAPK subfamilies, ERK is generally activated by growth factors and mitogens, while JNK and p38 MAPK are activated by cellular stresses and inflammatory cytokines [30]. In addition, JNK, p38 MAPK and NF-κB have been shown to be involved in age-related oxidative stress and chronic inflammation [32]. A previous study has shown that the myeloid cell-specific deletion of JNKs decreases the expression of macrophage-specific genes involved in the proinflammatory phenotype of LPS-stimulated bone-marrow-derived macrophages, suggesting a potential role in the treatment of inflammatory diseases by inhibiting JNK [31]. In the present study, our experimental results indicated that nordalbergin suppressed the phosphorylation of ERK, JNK and p38, indicating that nordalbergin effectively inhibited pro-inflammatory cytokines and mediators through inhibiting MAPKs signaling pathways in LPS-stimulated microglia.

Immune cells recognize the damage-associated molecular patterns (DAMPs) through receptors called pattern recognition receptors (PRRs). For the neurodegenerative diseases, the recognition of DAMPs via amyloid beta (Aβ) in AD, alpha-synuclein in PD, the mutant superoxide dismutase-1 (SOD-1) gene or transactive response DNA binding protein 43 (TDP-43) in amyotrophic lateral sclerosis (ALS) by PRRs leads to the activation of the resident brain immune cells such as microglia, resulting in neuroinflammation and neurodegenerative disease development and progression [33]. A recent study indicated that Aβ activates NLRP3 inflammasome via Toll-like receptor-4 in mouse microglia, resulting in the release of mature IL-1β [34]; additionally, aggregated Aβ also results in increasing phagocytosis and cytokine production and the promotion of neuroinflammation, consequently causing synapse loss and neurodegeneration [35]. The IL-1β pathway has been demonstrated as being essential for the synthesis of proinflammatory and neurotoxic factors in microglia, and the inflammasome, caspase-1 and IL-1β are critical for the recruitment of microglia to exogenous Aβ in the brain [36]. Targeting NLRP3 inflammasome activation has therefore been considered as a potential therapeutic target for the attenuation of AD progression [9,37]. Our experimental results indicated that nordalbergin inhibited NLRP3 inflammasome activation through suppressing the cleavage of caspase-1 and IL-1β and decreasing the secretion of IL-1β, suggesting that nordalbergin has the potential to attenuate NLRP3 inflammasome-induced neuroinflammation and impede neurodegenerative disease development and progression.

Oxidative stress that is generated from the unregulated production of ROS is thought to play an important role in neurodegenerative diseases [2]. When ROS is overproduced, it can cause oxidative deterioration by shifting the cellular redox balance toward an oxidative state, leading to cellular dysfunction and death [2]. Oxidative stress can exacerbate the progression of neurodegenerative diseases because of oxidative damage and its interaction with mitochondria [2,38], where the increase of ROS and intracellular Ca^2+^ would cause excessive Ca^2+^ influx into mitochondria, leading to mitochondrial dysfunction and subsequent neuronal loss [39]. Accordingly, it has been proposed that antioxidants might have the ability to alleviate the symptoms of neurodegenerative diseases by modulating oxidative stress in the biological environment [2]. Several antioxidants including superoxide dismutase, catalase, vitamin E and ascorbic acid have been investigated for their effects in attenuating neurotoxicity and promoting neuroprotection in neurodegenerative diseases [2]. Our experiments revealed that nordalbergin effectively suppressed the production of both intracellular and mitochondrial ROS in LPS-stimulated microglial cells, suggesting that nordalbergin might act as an effective antioxidant against oxidative stress.

## 4. Materials and Methods

### 4.1. Cell Culture

Murine BV2 microglial cells were purchased from the Food Industry Research and Development Institute (Hsinchu, Taiwan) and cultured in RPMI-1640 medium supplemented with 10% FBS, penicillin (100 U/mL) and streptomycin (100 U/mL) (Corning Inc., Corning, NY, USA) at 37 °C and passaged every 2–3 days to maintain growth.

### 4.2. NO Assay

The Griess assay was used to measure the level of accumulated nitrite (NO_2_^−^), a metabolite of NO, in the culture supernatant using the Griess reagent. A total of 2.5 × 10^4^ BV2 cells were seeded in 96-well plates and incubated overnight. Cells were then pretreated with different doses (0, 5, 10, 15 and 20 μM) of nordalbergin (purity > 98%, ChemFaces Wuhan, Hubei, China) for 30 min followed by LPS (from *E. coli* 0111: B4, Sigma Aldrich (St. Louis, MO, USA) treatment (0 or 1 μg/mL) for 24 h. Cell culture supernatant was collected and the production of NO was determined using the Griess reagent (Sigma Aldrich, St. Louis, MO, USA).

### 4.3. MTT Assay for Cell Viability

A total of 2.5 × 10^4^ BV2 cells were seeded in 96-well plates and incubated overnight. Cells were then pretreated with nordalbergin (0–20 μM) for 30 min, followed by 1 μg/mL LPS treatment for 24 h. Cell viability was analyzed by the MTT assay (Sigma, St. Louis, MO, USA).

### 4.4. ELISA

A total of 5 × 10^4^ BV2 cells were seeded in 96-well plates and incubated overnight. Afterward, cells were pretreated with nordalbergin (0–20 μM) for 30 min, and then treated with 1 μg/mL LPS for 24 h. The cell culture supernatant was harvested and analyzed to determine the cytokine expression of IL-6 and TNF-α by the ELISA (Thermo Fisher Scientific Inc., Waltham, MA, USA). For the measurement of IL-1β, cells were seeded in 96-well plates (5 × 10^4^ cells/well) and incubated overnight. After pretreatment with nordalbergin (0–20 μM) for 30 min, cells were treated with 1 μg/mL LPS for 47.5 h, followed by the treatment of ATP (5 mM) for 30 min. The supernatant was collected and analyzed using the ELISA (Thermo Fisher Scientific Inc., Waltham, MA, USA).

### 4.5. Western Blot Analysis

Total protein was obtained after cells were lysed by RIPA lysis buffer containing protease inhibitors and phosphatase inhibitors (Sigma Aldrich, St. Louis, MO, USA). Afterward, cell debris were removed by centrifugation at 4 °C, and the supernatants were quick-frozen. The BCA assay was then conducted to measure the concentration of protein lysates (Thermo Scientific Inc., Waltham, MA, USA). Subsequently, equal amounts of protein were separated via sodium dodecyl sulfate polyacrylamide gel electrophoresis and transferred to polyvinylidene fluoride membranes (EMD Millipore, Billerica, MA, USA). Membranes were then blocked using 5% skim milk or 0.1% bovine serum albumin (BSA) and then incubated with primary antibodies at 4 °C overnight. Afterward, membranes were incubated with horseradish peroxidase (HRP)-conjugated secondary antibody (1:5000) for 1 h at room temperature. Subsequently, membranes were washed three times with TBST or PBST, and chemiluminescent detection was performed to visualize the specific protein bands using a Bio-Rad ChemiDoc XRS^+^ system (Bio-Rad Laboratories, Inc., Hercules, CA, USA).

### 4.6. Flow Cytometry

A total of 5 × 10^5^ BV2 cells were seeded in 12-well plates and allowed to adhere overnight. Afterward, cells were pretreated with nordalbergin (0–20 μM) for 30 min following treatment of 1 μg/mL LPS for 24 h. For the detection of intracellular ROS, cells were stained with 1 μM H_2_DCFDA for 30 min using the H_2_DCFDA kit (Invitrogen, Carlsbad, CA, USA), and then detected at 492–527 nm by flow cytometry (Beckman Coulter, Elkin, NC, USA). For mitochondrial ROS detection, cells were stained with the MitoSOX kit and then detected at 510–580 nm by flow cytometry (Beckman Coulter, Elkin, NC, USA).

### 4.7. Statistical Analysis

GraphPad Prism 9 (GraphPad Software, San Diego, CA, USA) was used in all the statistical analyses. Data was analyzed by One-Way ANOVA followed by Tukey post-hoc tests. *p* < 0.05 was regarded as being statistically significant.

## 5. Conclusions

**Figure 6:**
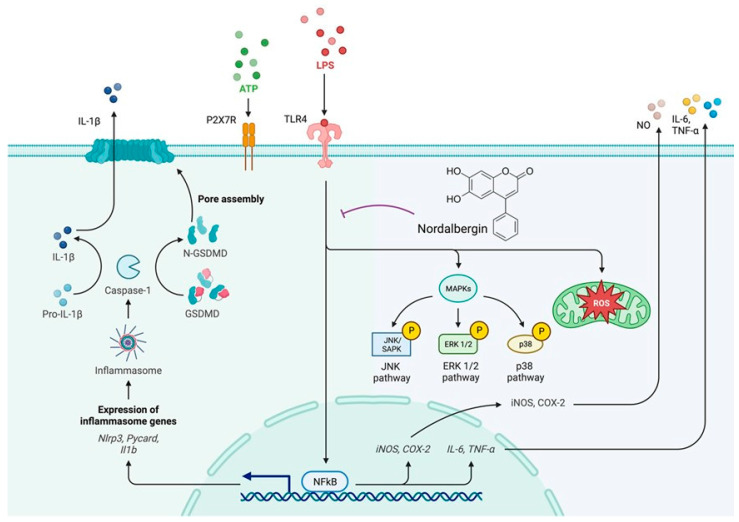
Nordalbergin exhibits anti-inflammatory and anti-oxidant properties in LPS-stimulated microglial BV2 cells. Our results demonstrated that nordalbergin decreases the secretion of NO, TNF-α, IL-1β and IL-6; represses the expression of iNOS and COX-2; suppresses the activation of MAPKs signaling pathways; attenuates the activation of NLRP3 inflammasome; and reduces the production of both intracellular and mitochondrial ROS by LPS-stimulated microglial BV2 cells. These results suggest that nordalbergin exerts anti-neuroinflammatory effects by attenuating MAPK signaling pathways, NLRP3 inflammasome activation and ROS production in LPS-stimulated BV2 microglia. The purple broken line means inhibition.

In summary, the present study is the first to demonstrate that nordalbergin suppresses the secretion of inflammatory mediators and cytokines and decreases the production of both intracellular and mitochondrial ROS through attenuating MAPK signaling pathways and NLRP3 inflammasome activation in LPS-stimulated microglial cells (Figure 6). These results suggest that nordalbergin exhibits anti-inflammatory and anti-oxidative effects, indicating its potential for managing neuroinflammation.

## Data Availability

Not applicable.
